# Negating Na‖Na_3_Zr_2_Si_2_PO_12_ interfacial resistance for dendrite-free and “Na-less” solid-state batteries†

**DOI:** 10.1039/d2sc05120f

**Published:** 2022-11-11

**Authors:** Rui Li, Daochuan Jiang, Peng Du, Chenbo Yuan, Xiaoyu Cui, Qichen Tang, Jian Zheng, Yecheng Li, Ke Lu, Xiaodi Ren, Shan Gao, Xiaowen Zhan

**Affiliations:** School of Chemistry and Chemical Engineering, School of Materials Science and Engineering, Institutes of Physical Science and Information Technology, Key Laboratory of Structure and Functional Regulation of Hybrid Materials of Ministry of Education, Anhui University Hefei 230601 P. R. China luke@ahu.edu.cn shangao@ahu.edu.cn xiaowen.zhan@ahu.edu.cn; Department of Materials Science and Engineering, University of Science & Technology of China Hefei 230026 P. R. China

## Abstract

Solid electrolytes hold promise in safely enabling high-energy metallic sodium (Na) anodes. However, the poor Na‖solid electrolyte interfacial contact can induce Na dendrite growth and limit Na utilization, plaguing the rate performance and energy density of current solid-state Na-metal batteries (SSSMBs). Herein, a simple and scalable Pb/C interlayer strategy is introduced to regulate the surface chemistry and improve Na wettability of Na_3_Zr_2_Si_2_PO_12_ (NZSP) solid electrolyte. The resulting NZSP exhibits a perfect Na wettability (0° contact angle) at a record-low temperature of 120 °C, a negligible room-temperature Na‖NZSP interfacial resistance of 1.5 Ω cm^2^, along with an ultralong cycle life of over 1800 h under 0.5 mA cm^−2^/0.5 mA h cm^−2^ symmetric cell cycling at 55 °C. Furthermore, we unprecedentedly demonstrate *in situ* fabrication of weight-controlled Na anodes and explore the effect of the negative/positive capacity (N/P) ratio on the cyclability of SSSMBs. Both solid-state Na_3_V_2_(PO_4_)_3_ and S full cells show superior electrochemical performance at an optimal N/P ratio of 40.0. The Pb/C interlayer modification demonstrates dual functions of stabilizing the anode interface and improving Na utilization, making it a general strategy for implementing Na metal anodes in practical SSSMBs.

## Abstract 2

A novel Pb/C interlayer is introduced on Na_3_Zr_2_Si_2_PO_12_ solid electrolyte, which offers perfect Na wettability, negates interfacial resistance, and allows *in situ* fabrication of “Na-less” anodes for stable solid-state Na-metal batteries.

## Graphical abstract

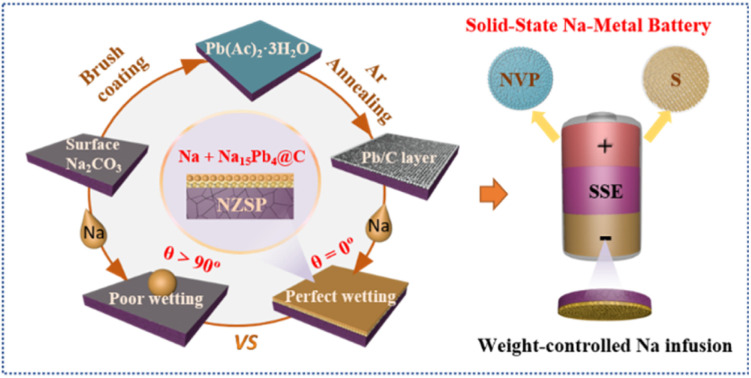

## 1 Introduction

The global trend towards decarbonization has transformed rechargeable batteries into one of the crucial enabling technologies for e-mobility and grid storage of intermittent solar and wind energies. Sodium-metal batteries (SMBs) with high energy density and abundant ingredient resources have been long considered to replace current lithium-based batteries, especially in large-scale grid applications.^1–6^ Carbonate-based liquid organic electrolytes used in most SMBs may arouse safety concerns such as leakage and short-circuits that may turn into fire and explosion.^6–8^ Solid electrolytes (SEs) can overcome the safety problems to a certain extent while still offering a wide operating temperature range, high energy density, and good electrochemical stability.^9,10^ Among SEs considered for solid-state SMBs, NASICON (Na superionic conductor)-type Na_1+*x*_Zr_2_Si_*x*_P_3−*x*_O_12_ (NZSP, 0 ≤ *x* ≤ 3), first reported by Goodenough *et al.* in 1976,^11^ has attracted much attention due to its high room-temperature ionic conductivity, wide electrochemical window, and excellent chemical stability towards Na.^7,11,12^ In recent years, strategies such as aliovalent doping and grain boundary tuning have been extensively pursued and proven efficient in further promoting the ionic conductivity of NZSP.^13–16^ For example, through Si/Zn co-doping and liquid-phase-aided sintering, Yao *et al.* achieved a record-high 5.27 mS cm^−1^ in Na_3.4_Zr_1.9_Zn_0.1_Si_2.2_P_0.8_O_12_.^16^ Concisely, the key challenge faced by NZSP-based solid-state SMBs is no longer improving conductivity but constructing stable and low-resistance interfaces between NZSP and electrodes, as such improving capacity utilization of electrodes towards high specific energy at the cell level.

Despite the good chemical/electrochemical stability between NZSP and Na, NZSP suffers from poor Na wettability due to the insufficient solid-to-solid physical contact (usually point-to-point) and the presence of the passivation layer. The insulating passivation layer (*i.e.*, Na_2_CO_3_) resulting from the spontaneous reaction between NZSP and H_2_O/CO_2_ in air not only introduces new interfacial resistance, but weakens the Na‖NZSP wettability thereby leading to huge interfacial contact resistance.^17^ The poor Na‖NZSP contact will deteriorate owing to volume changes during cell cycling, which eventually causes Na dendrite penetration into NZSP and thus a short circuit.

Existing strategies for improving NZSP‖Na anode interfacial wettability can be roughly classified into two categories: (1) removing surface passivation and (2) introducing sodiophilic interlayers. The former targets the removal of surface contamination such as Na_2_CO_3_, usually enabled by high-temperature annealing.^17,18^ However, this strategy could not achieve significant improvement in Na wettability. For example, the surface of heat-treated (450 °C) NZSP still exhibited a large wetting angle of 72.5°.^17^ The core concept of the second strategy is to enhance the interfacial wetting by exploiting the reaction (usually alloying) between the interlayer and Na. For instance, by forming the sodiophilic Na_15_Sn_4_ phase from the Na–Sn alloying reaction, Lu *et al.* achieved a high critical current density (CCD) of 2.5 mA cm^−2^ and a stable galvanostatic cycling for more than 500 cycles at 0.5 mA cm^−2^. Other interlayer materials such as SnS_2_, AlF_3_, and SnO_*x*_/Sn^19–21^ have been proven efficient. Although these reported interlayers show significantly improved molten Na wettability, the resistance associated with Na^+^ ion transport through the passivation layer itself and its interface with the interlayer might still exist, weakening the overall effect of the interlayer in reducing interfacial resistance. Therefore, comprehensive solutions simultaneously enabling surface passivation removal and interfacial wettability enhancement are urgently needed to minimize the interfacial resistance and stabilize the NZSP‖Na interface.

In addition to solving the interface contact problem, improving the Na anode utilization and thus the negative/positive capacity ratio (N/P ratio) in full cells is of equal importance for the implementation of high-energy-density solid-state SMBs. Similar to Li-metal batteries, the metallic Na anode in liquid-based batteries is always present in excess (*i.e.*, the use of hundred-micrometer-thick Na foil) so as to compensate the Na anode loss due to Na dendrite growth and continuous reaction with the liquid electrolyte. This greatly reduces the energy density and increases the cost. Solid-state systems are expected to reduce Na anode loss considering their chemically/electrochemically more stable anode interfaces.^22^ Unfortunately, most of the reported solid-state SMBs still exploited thick Na foil of various specifications in cell testing.^6–8^ As the Na anode loading in these studies was essentially unlimited, achieving notable cell-level energy density enhancement became impossible, which unarguably diminishes the practical value of such solid-state cells.^6–8^ Therefore, it is practically necessary to enable high Na utilization when designing anode interfaces for SSSMBs.

In this study, we propose a novel Pb/C modification to resolve the Na‖NZSP interfacial contact issue and improve the Na anode utilization. Through a simple brush-coating of lead acetate trihydrate (Pb(Ac)_2_·3H_2_O, or LAT) followed by annealing, not only is the surface passivation layer removed, but a perfect Na wetting (contact angle: 0°) can be achieved at 120 °C. The Na‖NZSP interfacial resistance was reduced from 391 Ω cm^2^ to 1.5 Ω cm^2^, and the corresponding Na symmetric cell could be stably operated at 0.5 mA cm^−2^ over 1800 h at 55 °C. With both Na_3_V_2_(PO_4_)_3_ (NVP) and S cathodes, solid-state cells using Pb/C@NZSP SEs showed significantly better cycling and rate performance than those with pristine NZSP. More importantly, *in situ* fabrication of weight-controlled Na anodes was realized for the first time to improve Na utilization, and excellent cyclability can be achieved in NVP-based cells at a low N/P ratio of 40.

## 2 Results and discussion

**Fig. 1:**
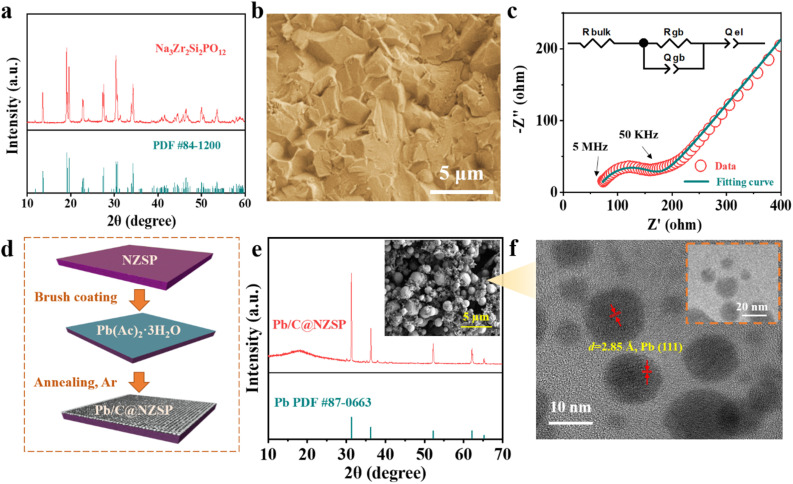
(a) XRD pattern of the NZSP powder. (b) Cross-section SEM image of the fractured NZSP pellet. (c) Impedance spectrum and the fitting results of a NZSP pellet measured at 25 °C using a Au‖NZSP‖Au cell configuration. The diameter and thickness of the pellet sample are, respectively, 10 mm and 0.8 mm. (d) A scheme illustrating the approach of the Na‖NZSP interface engineering strategy. (e) XRD pattern acquired at the surface of a Pb/C@NZSP pellet. The inset shows the corresponding surface SEM image. (f) TEM images of the Pb/C sample scratched from the Pb/C@NZSP surface.

The NZSP solid electrolytes were prepared following a conventional solid-state synthesis route. The X-ray diffraction (XRD) pattern of sintered NZSP pellets is shown in Fig. 1a, matching well with the standard pattern, except for two frequently observed minor peaks corresponding to the ZrO_2_ impurity.^20,23,24^ The fractural surface of NZSP pellets reveals a well-sintered microstructure featuring closely-packed cube-like grains and a high percentage of intragranular fractures, indicating the high density of NZSP (Fig. 1b).^25^ Impedance spectra of the NZSP pellets were measured at different temperatures as displayed in Fig. 1c and S1a, b.† The total ionic conductivity at 25 °C is calculated to be 6.1 × 10^−4^ S cm^−1^ with an activation barrier of 0.26 eV, both matching well with the reported data.^19–21,26–30^ Additionally, cyclic voltammetry measurement confirms that the NZSP is electrochemically stable within 7 V *vs.* Na/Na^+^ (Fig. S1c†).^31^ The simple and scalable Pb/C interlayer construction is schematically illustrated in Fig. 1d. Specifically, a saturated aqueous solution of LAT was brush-cast on the NZSP surface, followed by annealing in an Ar atmosphere (550 °C/5 h) to obtain the Pb/C-modified NZSP (hereinafter called Pb/C@NZSP). *In situ* Raman analysis deciphers the decomposition process of LAT when heated to 550 °C in Ar as shown in Fig. S2,† clearly disclosing the phase transformation of LAT to PbO, and finally to Pb/C.^32–34^ The existence of metallic Pb is confirmed using the XRD pattern acquired on the surface of a Pb/C@NZSP pellet (Fig. 1e). The SEM micrograph of the Pb/C@NZSP surface is displayed in the inset of Fig. 1e, revealing spherical Pb particles well distributed in the C matrix.^32^ Transmission electron microscopy (TEM) coupled with energy-dispersive X-ray spectrometry (EDX) further validated the presence of Pb nanoparticles surrounded by C, and the 2.85 Å reflection in the high-resolution TEM image outlined the location of Pb(111) planes (Fig. 1f and S3†).^35^ All this evidence strongly supports the successful construction of a porous Pb/C layer on the NZSP pellets.

**Fig. 2:**
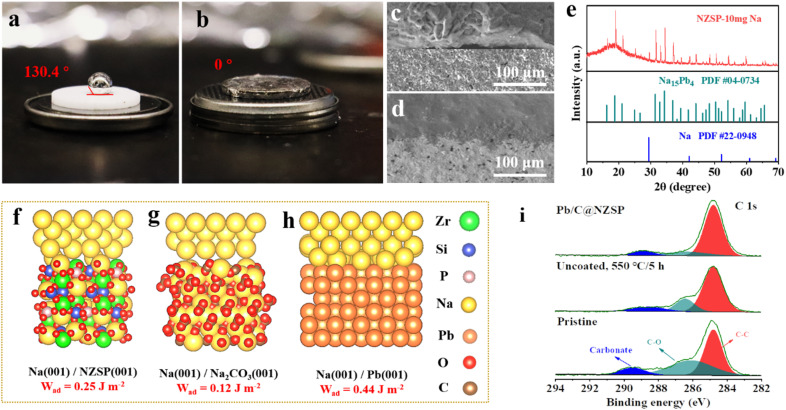
Contact-angle measurements of molten Na on (a) NZSP and (b) Pb/C@NZSP at 120 °C. SEM images of the cross-sections of (c) Na‖NZSP and (d) Na‖Pb/C@NZSP interfaces. (e) XRD pattern acquired on the surface of the Na-wetted Pb/C@NZSP. Theoretical structure simulation and work of adhesion (*W*_ad_) values for (f) Na(001)/NZSP(001), (g) Na(001)/Na_2_CO_3_(001) and (h) Na(001)/Pb(001). (i) High-resolution XPS spectra of C 1s acquired on the surfaces of the pristine NZSP, NZSP heated at 550 °C for 6 h in Ar, and Pb/C@NZSP.

To characterize the wettability of NZSP and Pb/C@NZSP by metallic Na, contact angle measurements were conducted at 120 °C. As shown in Fig. 2 and b, the untreated NZSP was barely wetted by molten Na, showing a large contact angle of 130.4°. In stark contrast, molten Na completely spread on the Pb/C@NZSP surface with a wetting angle of 0°. Considering both the wetting angle and testing temperature, this is unarguably the best Na wetting performance ever reported on NZSP or other solid electrolytes (*i.e.*, Na-β′′-alumina).^32,33^ As expected, the Pb/C@NZSP pellet exhibits intimate physical contact with Na, while obvious gaps can be found at the Na‖NZSP interface (Fig. 2c and d). Such poor interfacial contact between the pristine NZSP and Na was widely reported to result in large interfacial resistance and non-uniform Na deposition that eventually lead to dendrite growth.^20,36–38^ XRD was employed to analyze the composition of the Pb/C@NZSP surface wetted by 10 mg molten Na metal for identifying the reaction mechanism between the Na and Pb/C layer. As evidenced in Fig. 2e, part of the molten Na reacted with Pb to form the Na_15_Pb_4_ (PDF #04-0734) alloy, and the rest remained as Na metal (PDF #22-0948). To provide mechanistic insights into the significantly enhanced Na wettability, density functional theory (DFT) calculations were carried out to evaluate the interfacial work of adhesion, *W*_ad_, which represents the energy per unit area required to reversibly separate an interface into two free surfaces. That is, *W*_ad_ = (*E*_Na-slab_ + *E*_NZSP-slab_ − *E*_int_)/*A*, where *E*_int_ is the total energy of the heterostructure slab, and *E*_X-slab_ is the energy of an isolated slab with X = Pb(001), NZSP(001), Na_2_CO_3_(001). Fig. 2f–h show the optimal atomic structure of the low-energy interfaces, namely, Na(001)/NZSP(001), Na(001)/Na_2_CO_3_(001), and Na(001)/Pb(001), and the corresponding *W*_ad_ values were determined to be 0.25, 0.12, and 0.44 J m^−2^, respectively. According to the Young–Dupré equation, *W*_ad_ = *σ*_Na_ (1 + cos *θ*), where *σ*_Na_ = 0.2523 J m^−2^; a higher *W*_ad_ implies a smaller contact angle.^17^ Therefore, the trend revealed by the DFT calculations, that Na(001)/Pb(001) exhibits the highest *W*_ad_ and thus the smallest contact angle, is in line with the measurements shown in Fig. 2a and b. Moreover, based on the measured contact angle of 0°, a *W*_ad_ value of 0.5036 J m^−2^ can be estimated for Na(001)/Pb(001), which is close to the theoretical value of 0.44 J m^−2^, validating the feasibility of our DFT calculations. To exclude the interference by carbon and further affirm that the wetting enhancement is solely enabled by Pb, we also measured the contact angle between the molten Na and carbon-coated NZSP. A poor wetting behavior indicated by the large contact angle of 142° is shown in Fig. S4a.† The corresponding theoretical structure simulation is also provided in Fig. S4b.† The small *W*_ad_ value of 0.08 J m^−2^ estimated for Na(001)/C(001) again confirms the weak contact between molten Na and carbon. Obviously, the Na–Pb alloying effect is the key for Pb/C@NZSP to achieve perfect Na wettability and interfacial contact.

In addition to offering a remarkable sodiophilicity, the Pb/C surface modification also assists in the removal of the Na_2_CO_3_ passivation layer on NZSP pellets. In fact, we consider Na(001)/Na_2_CO_3_(001), shown in Fig. 2g, the more appropriate structure than Na(001)/NZSP(001) to model the interface between Na and the pristine NZSP. The reason is that NZSP tends to react with the moisture to form NaOH and eventually Na_2_CO_3_ after further reaction with CO_2_ in air,^17,18^ leaving the Na_2_CO_3_ passivation layer actually in contact with molten Na at a large contact angle (Fig. 2a). By substituting the observed contact angle of 130.4° in the Young–Dupré equation, a *W*_ad_ = 0.09 can be estimated, which is also closer to the theoretical value of Na(001)/Na_2_CO_3_(001) than to that of Na(001)/NZSP(001). To confirm the existence of the Na_2_CO_3_ passivation layer and its evolution during the Pb/C construction process, X-ray photoelectron spectroscopy (XPS) analysis was employed to examine the surface chemistry of the pristine NZSP, the unmodified NZSP after annealing, and Pb/C@NZSP. A major takeaway from Fig. 2i and S5† is that the surface carbonate can be significantly reduced by the annealing process in Ar, as evidenced by the decreased intensities in both the C 1s and O 1s spectra.^18,39^ Such decomposition of Na_2_CO_3_ on NZSP realized by high-temperature annealing was also confirmed by Huang *et al.*^17^ and Li *et al.*^18^ in their recent studies. To further verify that annealing LAT-coated NZSP can simultaneously remove the Na_2_CO_3_ passivation layer and form a Pb/C layer on the NZSP surface, we prepared a sample by heat treating a mixture of Na_2_CO_3_ and LAT at a weight ratio of 1 : 2 in Ar at 550 °C for 5 h. The XRD diffraction pattern (Fig. S6†) reveals mainly metallic Pb on the NZSP surface without Na_2_CO_3_ reflections despite the considerable portion of Na_2_CO_3_ present before annealing. Reducing elements such as Pb and C likely facilitate the reduction decomposition of Na_2_CO_3_ at high temperature. Such a strategy was, in fact, employed by Goodenough *et al.* to clean the surface lithium carbonate on Li_7_La_3_Zr_2_O_12_ solid electrolyte.^40^ In summary, considering all the above-mentioned results, it is safe to conclude that the approach of constructing a Pb/C layer not only improves the Na wettability and interfacial contact between Na metal and NZSP *via* Na–Pb alloying, but also helps remove the insulating Na_2_CO_3_ passivation layer, both of which are expected to reduce interfacial resistance, and homogenize Na^+^ ion deposition thereby inhibiting dendrite growth.

**Fig. 3:**
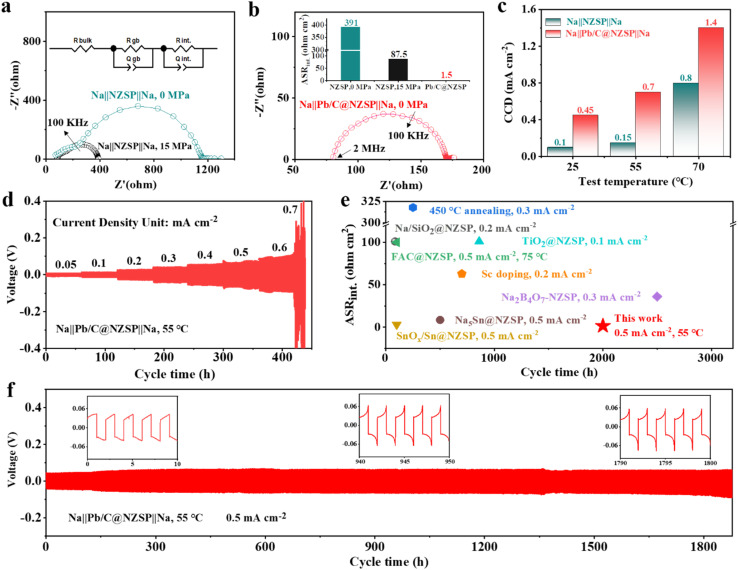
(a) EIS spectra of the Na‖NZSP‖Na symmetrical cell at 25 °C under a stacking pressure of 0 MPa or 15 MPa. (b) EIS spectra of the Na‖Pb/C@NZSP‖Na symmetrical cell at 25 °C under no stacking pressure. The ASR_int_ values of all symmetrical cells are compared in the inset of panel b. (c) The critical current density (CCD) values of the Na‖NZSP‖Na and Na‖Pb/C@NZSP‖Na symmetrical cells at 25, 55, and 70 °C. (d) Cycling profiles for the Na‖Pb/C@NZSP‖Na cell from 0.05–0.7 mA cm^−2^ at 55 °C. (e) Comparison of the ASR and cycle life of NZSP symmetrical cells modified by various processing strategies from the literature.^17,21,26,28,38,41–43^ (f) Long-term plating/stripping profiles of the Na‖Pb/C@NZSP‖Na cell at 0.5 mA cm^−2^ and 55 °C.

To unveil the electrochemical benefit of this strategy, EIS spectra of the corresponding Na‖NZSP‖Na and Na‖Pb/C@NZSP‖Na symmetrical cells were measured (Fig. 3a and b), with the detailed fitting results provided in Table S1.† Therein, *R*_b_, *R*_gb_, and *R*_int_ denote the contribution from the bulk (highest frequency intercept) and grain boundary (high-frequency semicircle) of NZSP and the Na‖NZSP interface (low-frequency semicircle), respectively.^44,45^ To facilitate the comparison with literature data, the interfacial resistance was converted to area specific resistance (ASR_int_) and plotted in Fig. 3b. The Na‖NZSP‖Na symmetric cell with pristine NZSP exhibits a large interfacial resistance of about 391 Ω cm^2^. By applying a stacking pressure of 15 MPa, a much smaller ASR of 87.5 Ω cm^2^ was achieved, which can be ascribed to the improved interfacial physical contact under external pressure.^46,47^ Remarkably, the Na‖Pb/C@NZSP‖Na cell free of stacking pressure afforded the lowest ASR_int_ of 1.5 Ω cm^2^, corresponding to a ∼261-fold reduction from that of the Na‖NZSP‖Na cell. This extremely low interface resistance is unarguably among the best records in the literature. Fig. S7† shows the EIS spectra of Na‖Pb/C@NZSP‖Na cells acquired under stacking pressures of 0 and 15 MPa. Clearly, the stacking pressure had little influence on the cell impedance, and was thus unnecessary for Pb/C@NZSP-based cells endowed with sufficient interfacial contact. In addition to the intimate physical contact (Fig. 2d), the ultrafast charge transfer kinetics at the interface can also be ascribed to the easier Na diffusion in the Na_15_Pb_4_ alloy than in metallic Na, a phenomenon widely confirmed in Li (*e.g.*, Li–Pb^34^) and Na (*e.g.*, Na–Sn^38^) alloy anodes.

The critical current density (CCD) is defined as the lowest current density at which cell shorting occurs, possibly due to Na dendrite penetration during cycling.^18,48^ Here, the CCD measurements were conducted for Na‖Pb/C@NZSP‖Na and Na‖NZSP‖Na symmetrical cells at 25, 55, and 75 °C (Fig. S8†), with the key trend summarized in Fig. 3c. Na‖Pb/C@NZSP‖Na shows significantly higher CCDs than Na‖NZSP‖Na at all tested temperatures. Notably, the CCD of Na‖Pb/C@NZSP‖Na reaches as high as 1.4 mA cm^−2^ at 70 °C, which is almost double that of Na‖NZSP‖Na (0.8 mA cm^−2^). As an example, the CCD of Na‖Pb/C@NZSP‖Na at 55 °C was double checked using an extended cycle number (*i.e.*, 30 cycles) at each current density (Fig. 3d), which arrived at the same number of 0.7 mA cm^−2^ (Fig. S8e†). In addition, the time-dependent EIS spectra (Fig. S9†) reveal little change in interfacial resistance, corroborating the superior interfacial stability of Na‖Pb/C@NZSP‖Na. When cycled at 0.2 mA cm^−2^/0.2 mA h cm^−2^ at room temperature, the Na‖Pb/C@NZSP‖Na cell exhibited an excellent cycling stability for over 600 h (Fig. S10a†). The time–voltage curve is consistently flat with a low voltage polarization of ∼28 mV, and the EIS spectra (Fig. S10b†) before and after cycling are identical except for a beneficial reduction in interfacial resistance, indicating a stable interface endowed with rapid Na^+^ transport. In contrast, the Na‖NZSP‖Na cell exhibited highly fluctuating overpotentials, and only sustained around 40 h before a complete short-circuit occurred (Fig. S10c†). Digital photo and SEM micrographs of the postmortem pellet surface clearly confirmed the rapid growth of sodium dendrites (Fig. S10d†) along the defects (*i.e.*, grain boundaries) due to inhomogeneous Na plating and excessive interfacial resistance.^49^ The superior cycling stability of Na‖Pb/C@NZSP‖Na is further demonstrated in Fig. 3f, where a remarkably long stability of >1800 h was achieved at a current density of 0.5 mA cm^−2^ at 55 °C. After the initial conditioning process, the Na‖Pb/C@NZSP‖Na cell maintained a constantly low overvoltage at around 60 mV as evidenced in the inset figures. Fig. 3e shows a comparison of ASR_int_ and cycle life of our work with some representative NZSP symmetrical cells reported in the literature using various interfacial engineering strategies. Considering both the extremely low interfacial resistance (1.5 Ω cm^2^) and ultralong cycle life (>1800 h) at a considerably high current density, our Na‖Pb/C@NZSP‖Na cell clearly afforded the best combined performance ever reported. To summarize, both experimental observation and theoretical calculations validated that the formation of the Na_15_Pb_4_ enables the perfect Na wetting and thus an ultralow interfacial resistance. Furthermore, the presence of the Na_15_Pb_4_ phase at the interface promotes Na diffusion kinetics, thereby delaying pores or void formation at the anode‖NZSP interface. These two merits have led to high critical current density and excellent cycling stability without dendrite growth of Na‖Pb/C@NZSP‖Na cells.^38,50^

**Fig. 4:**
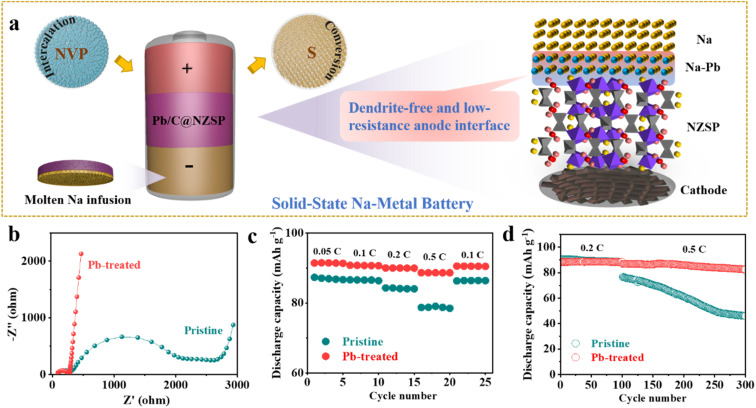
(a) Schematic illustration of the configuration of solid-state Na‖Pb/C@NZSP‖NVP and Na‖Pb/C@NZSP‖S batteries, highlighting the superiority of Pb-based anode interface engineering in homogenizing charge distribution and suppressing dendrite growth. (b) EIS spectra of Na‖NZSP‖NVP and Na‖Pb/C@NZSP‖NVP batteries measured before cycling. (c) Rate and (d) long-term cycling performances of Na‖NZSP‖NVP and Na‖Pb/C@NZSP‖NVP batteries.

As illustrated in Fig. 4a, taking advantage of the perfect Na wettability, Na anodes are *in situ* fabricated on NZSP solid electrolytes. NZSP-based solid-state batteries with low-resistance and dendrite-free anode interfaces were then demonstrated based on both intercalation-type NVP and conversion-type S cathodes. Initially, Na‖NZSP‖NVP cells with the pristine NZSP solid electrolyte and Na foil anode were demonstrated in parallel with Na‖Pb/C@NZSP‖NVP cells (see the “Experimental” section and Fig. S11† for the synthesis approach, XRD pattern, and morphology). Na loadings in both types of cells can be considered unlimited. Nyquist plots of the as-assembled NVP‖NZSP‖Na and NVP‖NZSP@Pb/C‖Na cells are shown in Fig. 4b, with the latter exhibiting much lower cell impedance owing to the more advantageous anode interface consisting of the Na_15_Pb_4_ alloy (Fig. 4a). As expected, the NVP‖NZSP@Pb/C‖Na cell supports obviously better rate performance than the NVP‖NZSP‖Na cell (Fig. 4c). The discharge capacity was 88 mA h g^−1^ at 0.5C and 90 mA h g^−1^ when returning to 0.1C for the NVP‖NZSP@Pb/C‖Na cell, while they were, respectively, 78 and 86 mA h g^−1^ for NVP‖NZSP‖Na. The charge–discharge curves at various rates are plotted in Fig. S12,† where the NVP‖NZSP@Pb/C‖Na cell shows much smaller polarization voltages. The long-term cyclability of both cells was examined by setting the cells at 0.2C for 100 cycles followed by 0.5C for 200 cycles as shown in Fig. 4d. The corresponding charge–discharge curves are presented in Fig. S13.† Both cells exhibit stable cycling profiles with identical capacities at 0.2C. When the rate was increased to 0.5C, the NVP‖NZSP@Pb/C‖Na cell maintained 82.5 mA h g^−1^ after 200 cycles, with a capacity retention rate of 79.9%. In stark contrast, the discharge capacity of NVP‖NZSP‖Na dropped immediately from 88.2 mA h g^−1^ to 76.5 mA h g^−1^ with the rate change, kept fading during cycling, and ended at only 49.7 mA h g^−1^. Cross-section SEM images were acquired on the anode interfaces of the cycled cells. As shown in Fig. S14a,† after disassembly, a large amount of Na was left on the spacer rather than on the NZSP pellet, indicating a loose anode interfacial contact of the Na‖NZSP‖NVP cell. Meanwhile, traces of Na penetration were revealed within the NZSP pellet, implying the possibility of Na dendrite growth and soft breakdown in the cell. In stark contrast, a sufficient amount of Na was retained for the Na‖Pb/C@NZSP‖NVP cell, as evidenced by both the digital and SEM images (Fig. S14b†). The clean, well-defined and intimately contacted anode interface aligns well with the superior rate and cycling performance of the Na‖Pb/C@NZSP‖NVP cell.

**Fig. 5:**
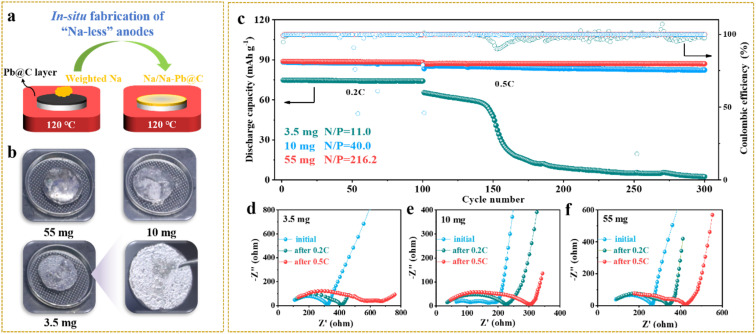
(a) Schematic illustration of the *in situ* fabrication of weight-controlled Na anodes on Pb/C@NZSP pellets, and (b) the corresponding digital photos. (c) The long-term cycling performances of Na‖Pb/C@NZSP‖NVP batteries with 3.5 mg (N/P = 11.0), 10 mg (N/P = 40.0), and 55 mg (N/P = 216.2) Na loadings. Nyquist plots of Na‖Pb/C@NZSP‖NVP full cells with limited Na loadings of (d) 3.5 mg, (e) 10 mg and (f) 55 mg measured before cycling, and after 0.2C and 0.5C cycling.

As we know, limiting the metallic anode excess, while rarely established yet in solid-state systems, is crucial to achieving high-energy-density solid-state batteries.^22^ To explore the pathways towards satisfactory battery performance at high Na utilization (or a low N/P ratio), we next assembled solid-state Na-metal batteries by paring weight-controlled Na anodes with NVP cathodes. Na anodes of 3.5, 10, and 55 mg were simply made by placing weighed Na chunks on the top of Pb/C@NZSP pellets kept at 120 °C to allow fast Na infusion (Fig. 5a). As can be seen in Fig. 5b, all anodes are fully spread. At 3.5 mg, a thin layer of silver-white Na tightly covered the pellet, while excessive Na hanging on the surface is evident as the Na mass increases. The cycling performances of Na‖Pb/C@NZSP full cells with different N/P ratios (Na weights) are shown in Fig. 5c and the corresponding voltage profiles are provided in Fig. S15.† Both cells at N/P ratios of 40.0 (10 mg) and 216.2 (55 mg) deliver very stable cycling performance at 0.2C and 0.5C, with coulombic efficiencies (CEs) consistently over 99.7%. The cell with 10 mg Na retained 82.4 mA h g^−1^ of its initial 85.4 mA h g^−1^ after 200 cycles at 0.5C, marking a high capacity retention of 96.5%. The full cell using 55 mg Na affords a slightly better performance by outputting 87.1 mA h g^−1^ after 200 cycles at 0.5C with no capacity fading. However, the full cell with 3.5 mg Na (N/P = 11.0) delivered a much lower initial capacity of 75.0 mA h g^−1^. Although stably operated at 0.2C for 100 cycles, it experienced a steep capacity drop to only 2 mA h g^−1^ after the rate rose to 0.5C, accompanied by fluctuating CEs.

EIS spectra were monitored during the cycling of all three cells (Fig. 5d–f) with the fitted cell resistances listed in Table S2.† The 3.5 mg cell exhibited an initial resistance of 330 Ω, which rose to 420 Ω after 100 cycles, and jumped to 700 Ω at the end. All these resistance values are the highest among the three cells, indicating that the 3.5 mg cell had the worst anode interface kinetics consistent with its lowest capacity at both rates (Fig. 5c). In contrast, the 10 mg and 55 mg cells show much lower and more stable cell resistances throughout the cycling, which is consistent with the excellent cyclability and high capacity output. To acquire more evidence, a postmortem morphology analysis was conducted. Fig. S16a–c† show SEM micrographs acquired on Na anode surfaces. While the 55 mg cell still had full Na coverage on NZSP solid electrolyte after cycling, the anode surface of the 3.5 mg cell manifested major Na depletion, leaving most of the Pb/C@NZSP surface naked. The cross-section SEM images acquired on the Na‖Pb/C@NZSP interfaces reveal a similar trend (Fig. S16d–f†). At a first glance, all three cells maintained good interfacial contact benefiting from the excellent Na wetting. However, the Na coverage clearly reduces with lowering the Na loading, and the 3.5 mg cell exhibited notable Na scarcity and inhomogeneity. The morphologies of cycled NVP cathodes were also examined as shown in Fig. S16g–i,† where no obvious difference exists among the three cathodes. It is hypothesized, based on the galvanostatic cycling, EIS and SEM analysis, that 3.5 mg is too less to achieve full Na penetration into the Pb/C porous layer, resulting in the insufficient active area for charge-transfer reactions at the interface region.^34^ The sluggish kinetics will induce increasingly inhomogeneous Na plating/stripping with cycling, especially at higher current rates (*e.g.*, 0.5C), causing dendrite growth and even a “soft” short circuit as implied by the up-and-down CEs and Na depletion at the far-end.^22,38,51^

The idea of tuning Na loading was also extended to S cathodes of greater potential in high-energy-density SSSMBs (see the “Experimental” section and Fig. S17† for the cathode preparation details). As shown in Fig. S18a,† the Na‖Pb/C@NZSP‖S cells deliver a superior initial capacity of 828.8 mA h g^−1^, overpowering that of the Na‖NZSP‖S cell (212.5 mA h g^−1^). Moreover, such a high initial capacity (>800 mA h g^−1^) is still accessible even when we cut Na loading to reach an N/P ratio of around 40 (Fig. S18b†). Table 1 summarizes the electrochemical performance of some representative SSSMBs based on NZSP solid electrolytes. Although reducing the Na metal amount for practical SMBs is of great importance, few research groups have laid eyes on it, mostly because of the great challenge in controlling Na loading while ensuring perfect interfacial contact. In this work, we have successfully demonstrated NVP- and S-based solid-state SMBs with remarkable cycling performance at unprecedentedly low N/P ratios (*i.e.*, 40), representing an important step towards practical high-energy-density batteries.

**Table 1:** Literature review of some representative solid-state Na-metal batteries based on NASICON-type Na_3_Zr_2_Si_2_PO_12_ (NZSP) solid electrolytes

Battery structure	N/P ratio	Current rate	Capacity (mA h g^−1^)	Mass loading (mg cm^−2^)	Cycle life	Ref.
Na||trilayer NZSP||NVP	Unlimited Na	1C	96.7	—	450	52
Na||Pr-NZSP||NVP	Unlimited Na	0.5C	109.1	∼1	100	24
Na||Sc-NZSP||NVCP	Unlimited Na	100 mA g^−1^	105	2–3	100	42
Na||AlF_3_@NZSP||NVP	Unlimited Na	1C	133.1	1.5	100	19
Na||annealed NZSP||NVP	Unlimited Na	1C	106	1	100	18
Na||SnS_2_@NZSP||NVP	Unlimited Na	1C	104	1	100	20
Na||NZSP||NaCrO_2_	Unlimited Na	5C	102.6	1.5	400	53
Na||NZSP-Na_2_B_4_O_7_||NVCP	Unlimited Na	100 mA g^−1^	100	2–3	195	43
Na||SnO_*x*_/Sn-NZSP||NVP	Unlimited Na	1C	103.1	N/A	100	21
Na||Pb/C@NZSP||NVP	11.0	0.2C	74.7	3.15	100	This work
Na||Pb/C@NZSP||NVP	40.0	0.2C; 0.5C	88.2	2.52	100; 200	This work
Na||Pb/C@NZSP||NVP	216.2	0.2C; 0.5C	88.9	2.52	100; 200	This work
Na||Pb/C@NZSP||S^a^	∼40	60 mA g^−1^	828.8	∼1	25	This work

a All batteries in the list were operated at room temperature, except for our Na–S battery that was operated at 60 °C.

## 3 Conclusion

In summary, the present work introduces a novel Pb/C interlayer for NZSP solid electrolytes by employing a simple and scalable brush-coating process combined with annealing. Not only is the surface passivation layer removed, but a perfect Na wetting (contact angle: 0°) can be achieved on Pb/C@NZSP at a record-low temperature of 120 °C. Physicochemical characterization and DFT calculations elucidated the wetting enhancement mechanism essentially based on the Na–Pb alloying reaction. Moreover, the formation of the Na_15_Pb_4_ phase promotes Na diffusion kinetics, thereby delaying pore or void formation at the anode‖NZSP interface to inhibit dendrite growth. As a result, the Na‖NZSP interfacial resistance was reduced from 391 Ω cm^2^ to 1.5 Ω cm^2^. The corresponding Na symmetric cell affords a high CCD of 1.4 mA cm^−2^ at 70 °C, and could be stably operated at 0.5 mA cm^−2^ over 1800 h at 55 °C. More importantly, the highly sodiophilic Pb/C interlayer can function as a Na host thereby enabling *in situ* fabrication of weight-controlled Na anodes with a low-impedance interface with NZSP. Benefiting from this, we for the first time demonstrate SSSMBs with concise control of the practically relevant N/P ratio. Markedly, the Na_3_V_2_(PO_4_)_3_ full cells with a low N/P ratio of 40.0 can be stably operated for 300 cycles with negligible capacity fade. Overall, our Pb/C interlayer strategy offers a comprehensive solution to handling the interfacial contact issue while enabling high Na utilization. The findings presented here absolutely represent a significant step towards developing practical high-energy-density SSSMBs.

## Data availability

Experimental procedures, compound characterization, and additional data have been included in the ESI.†

## Author contributions

R. Li prepared and characterized the materials and conducted electrochemical tests. D. Jiang performed theoretical calculations. P. Du, C. Yuan, X. Cui, J. Zheng, and Q. Tang assisted in materials synthesis and data analysis. Y. Li carried out SEM characterization studies. K. Lu supervised full cell tests. R. Li, K. Lu and X. Zhan wrote the paper. K. Lu, X. Ren, S. Gao, and X. Zhan revised the manuscript and discussed the results. X. Zhan and S. Gao conceived and supervised the project.

## Conflicts of interest

There are no conflicts of interest to declare.

## Supplementary Material

SC-013-D2SC05120F-s001
