# Supplementary material for: Negating Na‖Na3Zr2Si2PO12 interfacial resistance for dendrite-free and “Na-less” solid-state batteries
Source: Chem Sci. 2022 Nov 11;13(47):14132–40. doi: 10.1039/d2sc05120f (PMC9728568; doi:10.1039/d2sc05120f)
Supplement: SC-013-D2SC05120F-s001 [file SC-013-D2SC05120F-s001.pdf]

Electronic Supplementary Material (ESI) for Chemical Science.
This journal is © The Royal Society of Chemistry 2022

Supporting Information

Negating Na||$Na_3Zr_2Si_2PO_{12}$ Interfacial Resistance for Dendrite-Free and "Na-Less" Solid-State Batteries

Rui Li[#], Daochuan Jiang[#], Peng Du, Chenbo Yuan, Xiaoyu Cui, Qichen Tang, Jian Zheng, Yecheng Li, Ke Lu[*], Xiaodi Ren, Shan Gao[*], Xiaowen Zhan[*]

Experimental Section

Preparation of $Na_3Zr_2Si_2PO_{12}$ Solid Electrolytes. The $Na_3Zr_2Si_2PO_{12}$ (NZSP) samples were synthesized via a conventional solid-state reaction method. The initial materials were Na_2CO_3 (99.9%, Aladdin), SiO_2 (99.9%, Aladdin), ZrO_2 (99.99%, Aladdin), $NH_4H_2PO_{12}$ (99.99%, Aladdin). 20% excess of Na_2CO_3 and $NH_4H_2PO_{12}$ were used to compensate for the Na and P volatilization during the high-temperature calcination process. Stoichiometric amounts of the above chemicals were mixed and ground at 300 rpm for 24 h using a planetary ball mill (YXQM-1L, MITR, Changsha, China) with 5 and 10 mm ZrO_2 balls and ethanol solvent in a ZrO_2 jar. Subsequently, the ball-milled mixture was dried in a vacuum oven at 80 °C for 4 h before it was preheated at 600 °C for 4 h and calcined at 1150 °C for 4 h in air. The calcined powder was ground in an agate mortar, and then ball-milled again for 2 h at 450 rpm. The obtained powder was dried and pressed uniaxially (5 metric tons, YLJ-15T-LD, MTI Corp., Hefei, China) to form green pellets of 10 mm diameter and 1 mm thickness, which were further densified using a cold isostatic press (YLJ-CIP-15, MTI Corp., Hefei, China) under 200 MPa. Finally, the pellets were sintered at 1200 °C for 6 h in an alumina crucible in air. In order to prevent Na and P loss during sintering, all NZSP pellets were placed over a powder bed of identical composition in the bottom of the crucible to prevent reactions with alumina and covered by the same powder to reduce Na/P loss during sintering. All heating and cooling rates were set at 5 °C/min. To avoid reactions with moisture or carbon oxides in the air, the sintered NZSP pellets were transferred to and stored in an Ar-filled glovebox for further use.

Pb/C surface modification. 10 μL aqueous solution of saturated $Pb(Ac)_2 \cdot 3H_2O$ was drop on the surface of a NZSP pellet and distrusted evenly using a brush. For samples used in symmetrical cell testing, both sides of NZSP pellets were coated with $Pb(Ac)_2 \cdot 3H_2O$ using the same method. After drying, the coated NZSP pellets were transferred into a tube furnace and heated at 550 °C for 5 h under Ar flow. The carbon-coated NZSP was prepared by following the same protocol but with a $NaAc \cdot 3H_2O$ precursor. To reduce exposure to air, the quartz tube containing the treated pellets was transferred into a glove box immediately after the samples were cooled to 90 °C.

Preparation of NVP/C cathode. In a typical synthesis, 15 mmol Na_2CO_3 (99.9%, Aladdin), 10 mmol V_2O_5 (99%, Energy Chemical), 30 mmol $NH_4H_2PO_4$ (99.99%, Aladdin), and 1 g $C_6H_{12}O_6$ (99%, Sinopharm Chemical Reagent Co., Ltd.) were ball-milled for 12 hours at 300 rpm in ethanol using an alumina jar. The obtained mixture was uniaxially pressed into a pellet that was subsequently annealed at 450 °C for 4 h under Ar flow. After cooling, the precursor pellet was crushed and ball-milled again in ethanol at 300 rpm for 12 hours using an alumina jar. Finally, the NVP/C sample was produced by calcinating the dry precursor powders at 800 °C for 24 h in Ar. To fabricate NVP/C electrodes, a slurry of the NVP/C powder, Super P (MTI Corp., Shenzhen), and PVDF (Kynar HSV 900) at a weight ratio of 70: 20: 10 was cast onto carbon-coated Al foil (MTI Corp., Shenzhen, 18 mm in thickness). The electrode material loading is about 2-3 mg cm^{-2}.

Preparation of S cathode. The Fe_3C- and nitrogen-doped carbon at activated porous carbon cloth (Fe_3C-NC@ACC) matrix was prepared using a previously reported method.[1] The activated porous carbon cloth was prepared by a simple alkaline activation treatment on commercial carbon cloth. Typically, a piece of ACC was immersed in 40 mL DI water that contains 0.5 mL pyrrole monomer and 0.8 mL hydrochloric acid solution. 1.0 g of potassium ferricyanide was added into 10 mL DI water under stirring. After cooling down to about 4 °C,

both solutions were mixed together and kept at 4 °C for 4 h. The resultant carbon cloth was washed by DI water and dried at 60 °C, followed by annealing at 800 °C for 2 h in N_2. Subsequently, sulfur was loaded on the carbon composite scaffold according to a simple inside encapsulation method to obtain S/Fe_3C-NC@ACC. The carbon matrix was immersed in the sulfur/CS_2 solution until the mixture was completely dry. Then, the mixture was transferred into a sealed glass bottle filled with argon and heated at 155 °C for 10 h. The sulfur contents in the composite sample were about 1.0 mg/cm^2. Finally, the sulfur cathode in solid state was prepared by mixing 80 wt% S/Fe_3C-NC@ACC and 20 wt.% PEO10-NaFSI in acetonitrile. The PEO10-NaFSI was prepared by mixing 40 wt.% NaFSI and 60 wt.% PEO (M.W.: 5,000,000, Sigma-Aldrich) in acetonitrile and stirred at 60 °C for at least 24 h in Ar-filled glove box.

Physicochemical characterizations. XRD patterns were collected using an X-ray diffractometer (SmartLab9 KW) at a scan rate of 10° min^{-1} in the 2θ range of 10-80°. The microstructural and composition analysis of the powders and fractured cross-sections of NZSP pellets were conducted on a field emission scanning electron microscope (SEM, Hitachi S4800) equipped with an energy dispersive spectrometer (EDS). Surface chemical states of NZSP pellets were identified by X-ray photoelectron spectroscopy (XPS, PHI QuanteraII) on an X-ray photoelectron spectrometer with Al Kα as the X-ray source, and all binding energies of samples were corrected by referencing the C 1s peak to 284.8 eV. An *in-situ* heating kit (Beijing Scistar Technology) was used for the *in-situ* Raman study on the decomposition process of $Pb(Ac)_2 \cdot 3H_2O$ coated on NZSP pellets. Raman spectra were collected on a Via-Reflex Raman spectrometer (HORIBA XploRA PLUS, Japan) equipped with a 50 × -LWD objective and laser at 638 nm with 8 mW laser power. The time acquisition was 10 s and 10 scans were recorded to improve the signal-to-noise ratio. For contact-angle measurements, NZSP pellets were preheated on a hot plate at 120 °C inside the glovebox, and a small piece of Na metal (oxidation layer pre-removed) was placed on top of NZSP pellets to allow the observation of wetting

phenomena.

Electrochemical Testing. The ionic conductivity of NZSP pellets was determined by AC impedance measurements conducted on a Gamry REF 600+ potentiostat/galvanostat (Gamry, USA) using an Au||NZSP||Au configuration from 5 MHz to 1 Hz with an amplitude of 10 mV. For the assembly of Na||NZSP||Na symmetric cells, two Na disks (diameter: 9 mm) were hand-pressed on both sides of the NZSP pellet. For the assembly of Na||Pb/C@NZSP||Na cells, Na was preloaded on both sides of the Pb/C@NZSP pellet by a molten Na infusion process. All Na symmetric cells were assembled using a pressure-controllable Swagelok-type cell holder (EQ-PSC, MTI Corp.). The stacking pressure control was enabled with the help of a uniaxial press equipped with a pressure sensor (YLJ-5T, MTI Corp.). To be specific, the pressure sensor reads the mass load (m) in kg, which can be converted to the pressure applied on the NZSP pellet based on $P=mg/A$, where A is the effective area of the pellet. For instance, a stacking pressure of 15 MPa used in the present work was estimated based on the reading load (120 kg) and the pellet area (0.8 cm^2):

$$P = (120 \text{ kg} * 10 \text{ m/s}^2) / 0.8 \text{ cm}^2 = 1500 \text{ N/cm}^2 = 15 \text{ MPa}.$$

The impedance measurements were carried out over a frequency range of 5 MHz to 1 Hz with a 10 mV amplitude. All EIS spectra were analyzed and fitted by a Z-view software. For NVP-based full cells, 10 μL liquid electrolyte (1 M $NaClO_4$ in EC/DMC (1:1) + 5% FEC) was used to improve the cathode interface contact. For S-based solid-state cells, S cathodes were assembled directly with the NZSP solid electrolyte without liquid electrolyte. For NZSP based full cells, a Na disk was hand press on the anode side; for Pb/C@NZSP based full cells, Na anode was fabricated by pre-infusing molten Na into the Pb/C modified side. All full cells were assembled in an argon-filled glove box ([H_2O] < 0.1 ppm, [O_2] < 0.2 ppm) at room temperature using a coin-cell configuration and cycled using a NEWARE-BTS battery tester (NEWARE, Shenzhen). An incubator (Neware CT-9004, 25 ºC) was used for temperature control unless

mentioned otherwise.

Density Functional Theory (DFT) Calculations. All DFT calculations were performed with the VASP code,[2] Projector Augmented Wave (PAW) methods were used for the pseudopotentials.[3] The generalized gradient approximation (GGA)with the Perdew-Burke-Emzerhof (PBE) functional was employed to describe the exchange correlation energy.[4] The energy cutoff was set to 450 eV. The convergence criterion for the electronic self-consistency loop and atomic forces was set to 10^{-5} eV and 0.05 eV $Å^{-1}$, respectively. To simulate the interface of Na/Pb, Na/NZSP, Na/Na_2CO_3 and Na/C, the lattice constant of four layers of Na (001) slab was adjusted to adapt the dimensions of Pb (001), NZSP (001), Na_2CO_3 (001) and C (001). The number of atoms in these interfaces and the lattice parameters for the interfaces are listed in **Table S3**. A vacuum region of 15 Å was set along the z direction and the back half atoms that are farthest away from the interface in each slab were fixed for all the heterostructure geometry optimizations. A 1×1×1 k-point mesh was used for the Brillouin Zone sampling to speed the optimization process.

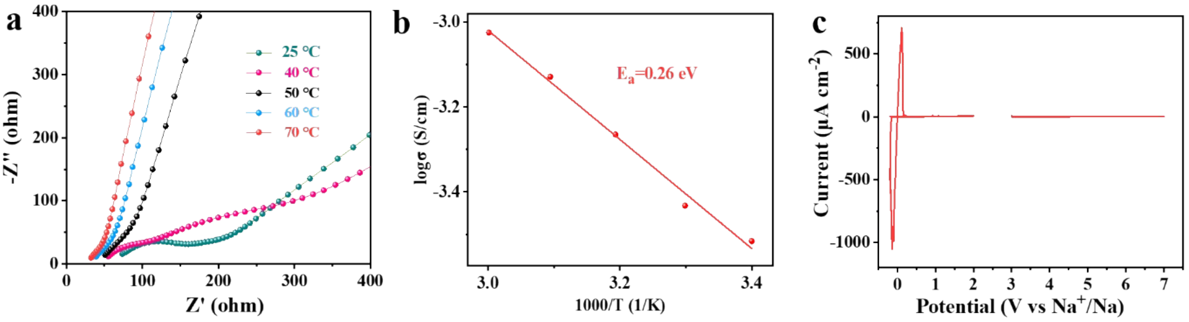

Figure S1. (**a**) Evolution of impedance spectra with temperature, and (**b**) the corresponding Arrhenius plot of the NZSP pellet. (**c**) CV curves of a Na||NZSP||Au cell within -0.2-2.0 V (0.3 mV s^{-1}) and 3.0-7.0 V (0.05 mV s^{-1}).

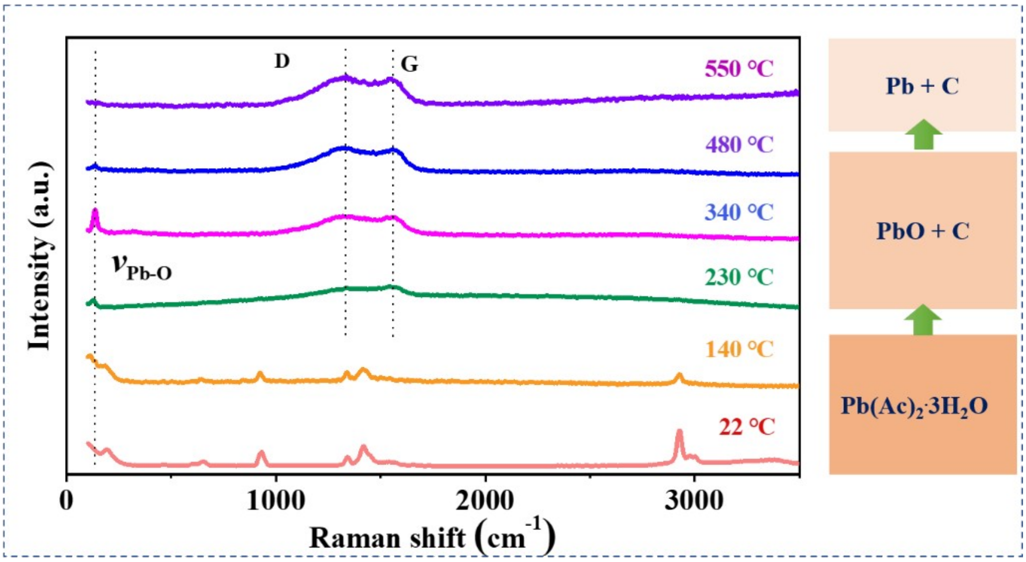

Figure S2. In-situ Raman spectra for the annealing process of $Pb(Ac)_2 \cdot 3H_2O$ conducted from 22 °C to 550 °C.

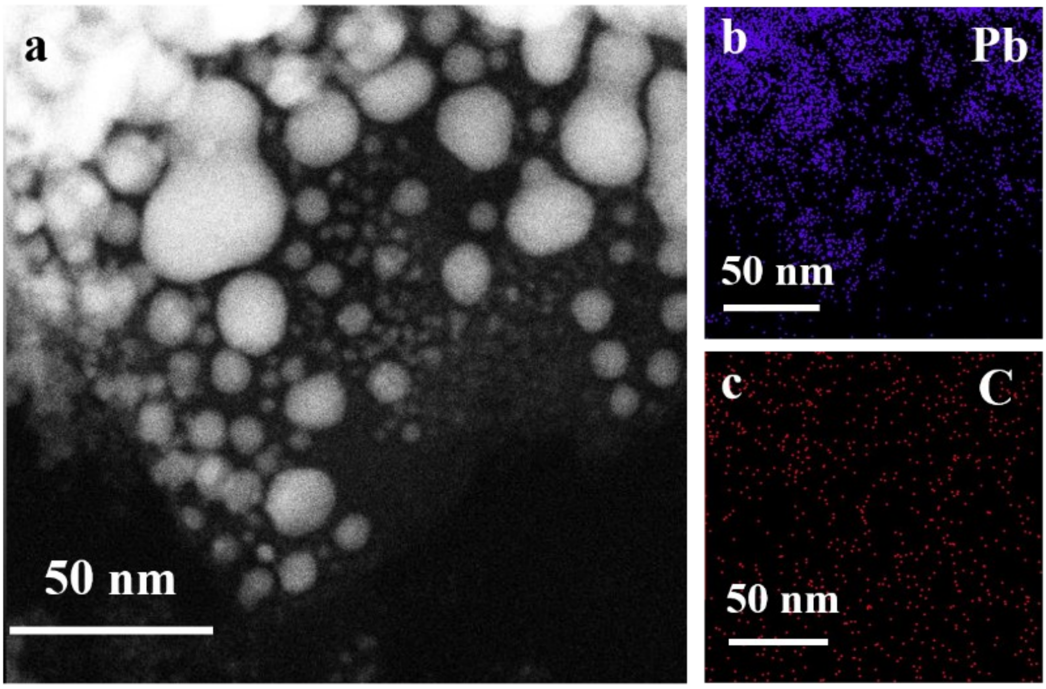

Figure S3. TEM and EDX images of the Pb/C sample scratched from the Pb/C@NZSP surface.

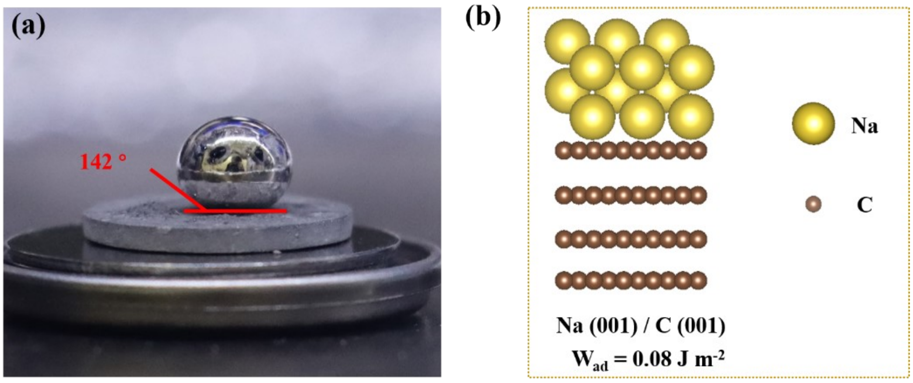

Figure S4. (a) Contact-angle measurement of molten Na on the carbon-coated NZSP surface. **(b)** Theoretical structure simulation and work of adhesion (W_{ad}) values for the Na(001)/ C(001) interface.

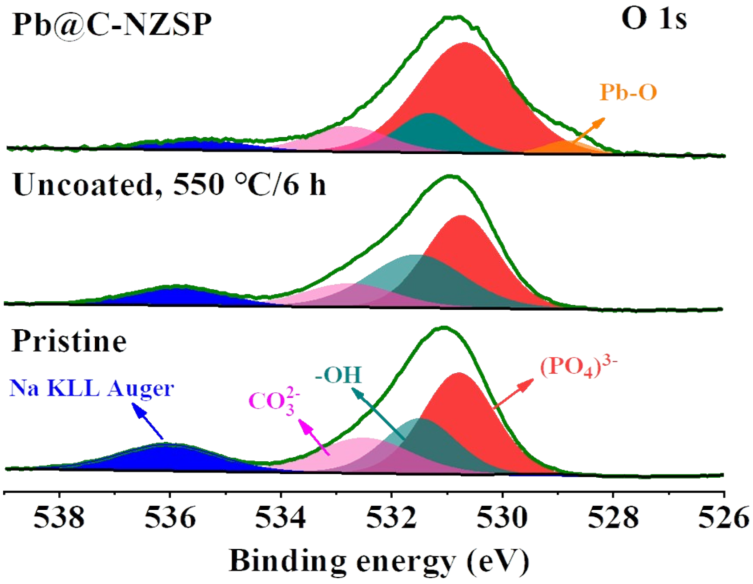

Figure S5. High-resolution XPS spectra of O 1s acquired on the surfaces of the pristine NZSP, NZSP heated at 550 °C for 5 h in Ar, and Pb/C@NZSP.

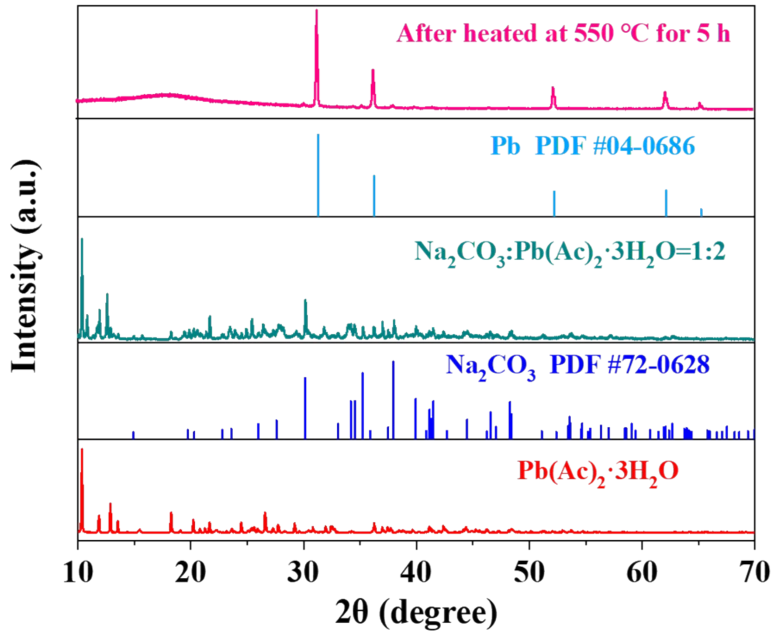

Figure S6. XRD diffraction patterns of the powder mixture (the weight ratio of Na_2CO_3 to $Pb(Ac)_2{\cdot}3H_2O$ is 1:2) before and after annealing at 550 °C for 5 h under Ar flow.

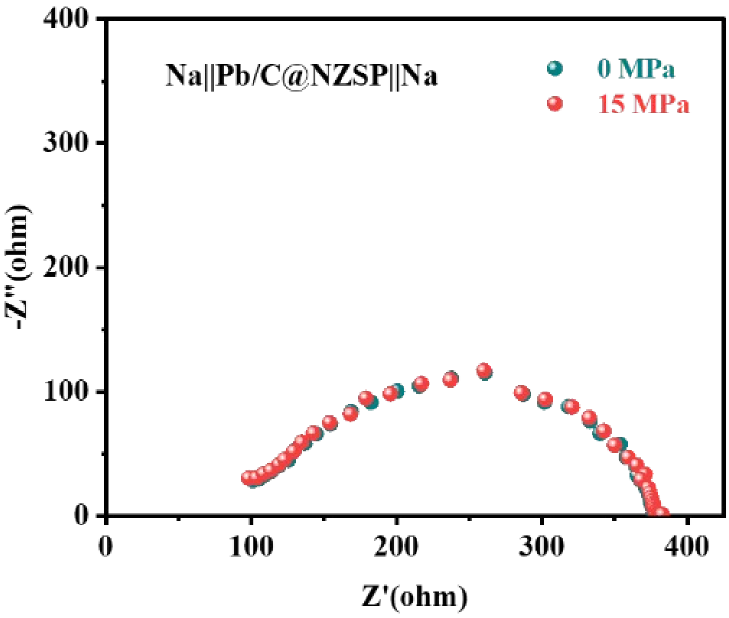

Figure S7. EIS spectra of Na||Pb/C@NZSP||Na symmetrical cells measured at 25 ºC with different stacking pressures of 0 MPa and 15 MPa.

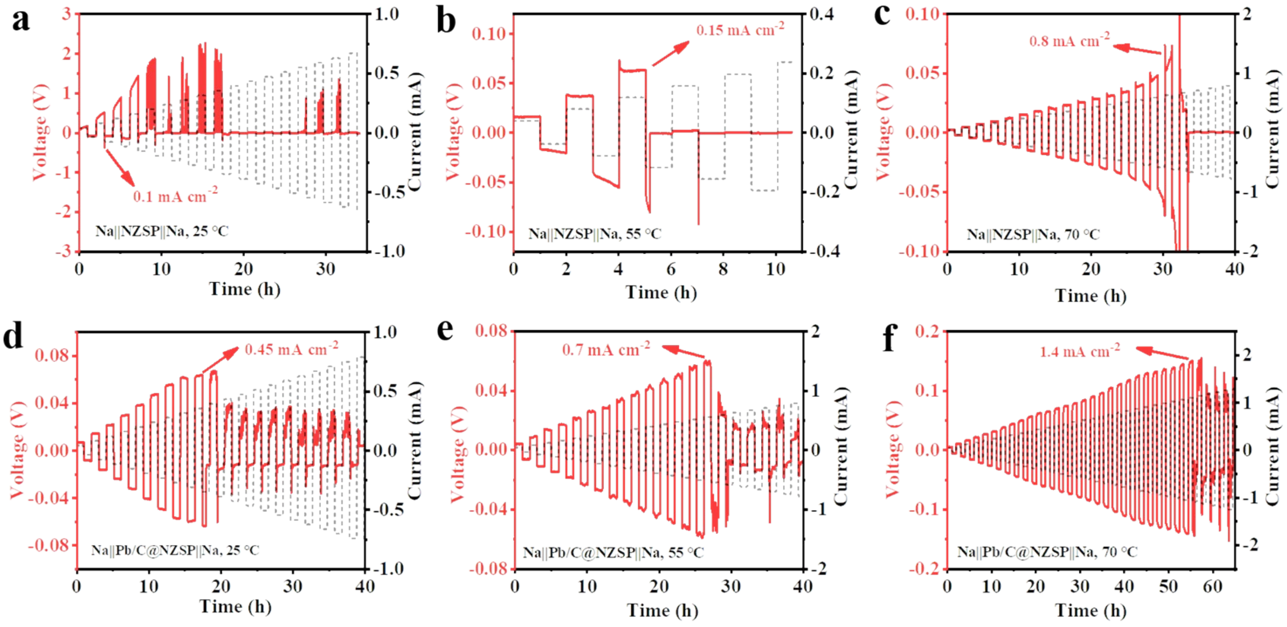

Figure S8. The CCD measurement profiles of **(a-c)** Na||NZSP||Na and **(d-f)** Na||Pb/C@NZSP||Na symmetric cell at 25 °C, 55 °C, and 70 °C.

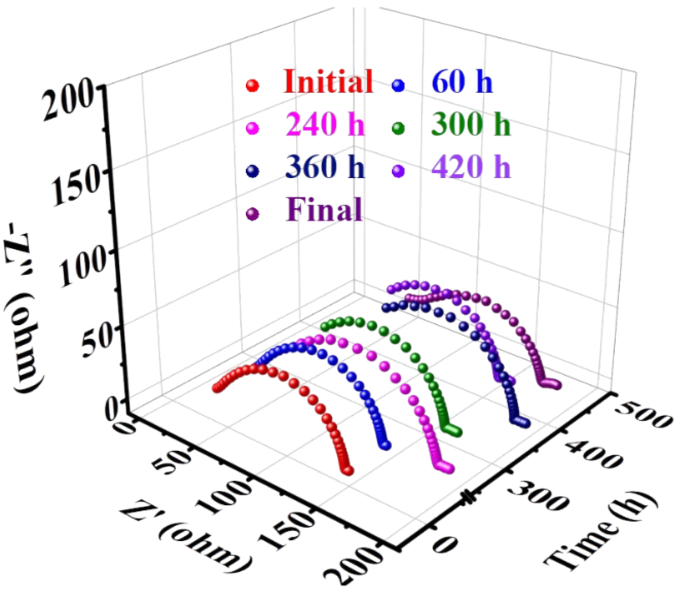

Figure S9. EIS spectra for the Na||Pb/C@NZSP||Na cell from 0.05-0.7 mA cm^{-2} at 55 °C.

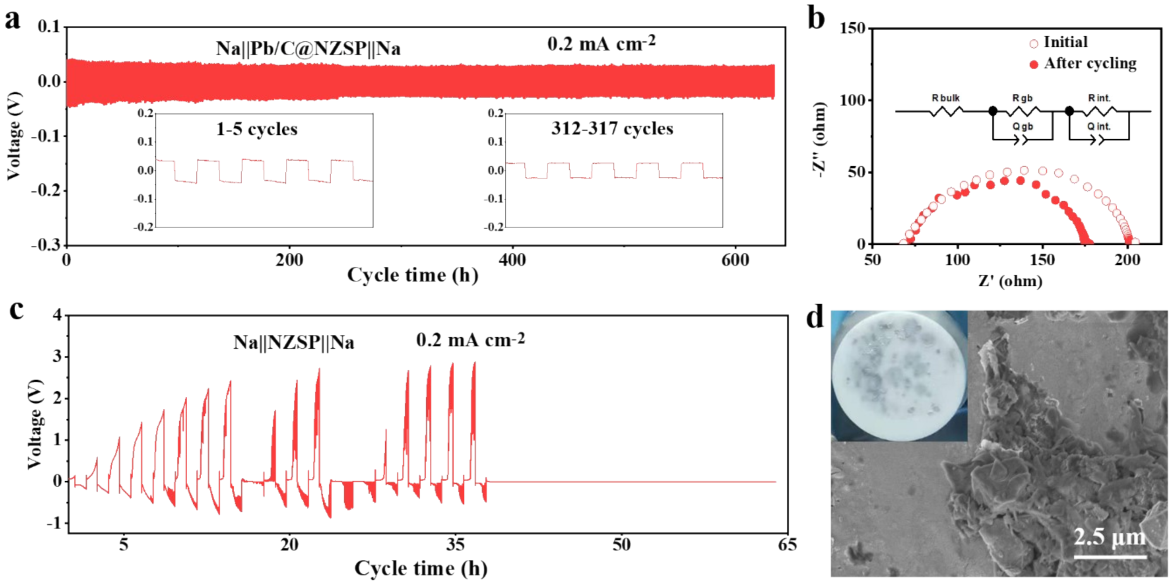

Figure S10. (**a**) Galvanostatic cycling curves of the Na||Pb/C@NZSP||Na symmetrical cell at 0.2 mA cm^{-2} at 25 ºC. (**b**) EIS spectra of the Na||Pb/C@NZSP||Na symmetrical cell before and after cycling at 0.2 mA cm^{-2}.(**c**) Galvanostatic cycling curves of the Na||NZSP||Na symmetrical cell at 0.2 mA cm^{-2} at 25 ºC. (**d**) SEM image and digital photo taken on the NZSP pellet surface after the cell failure originating from dendrite growth.

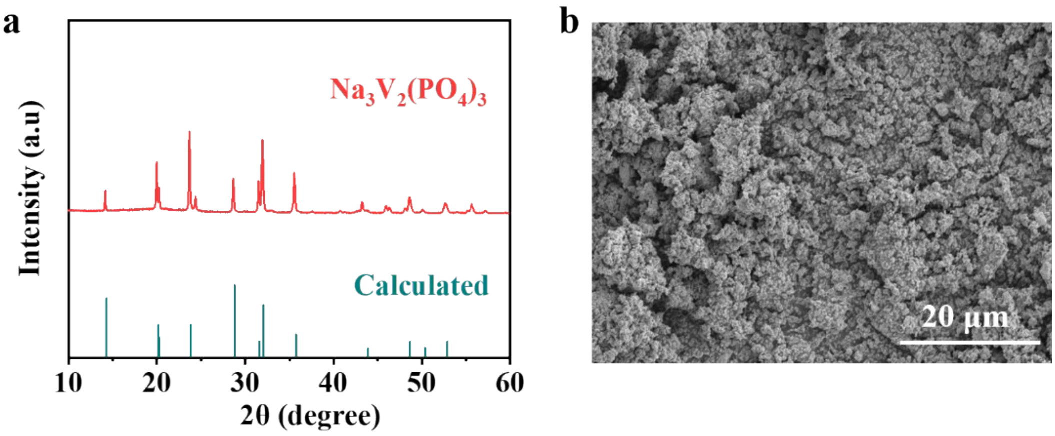

Figure S11. (**a**) XRD pattern and (**b**) SEM image of as-synthesized $Na_3V_2(PO_4)_3$ powders.

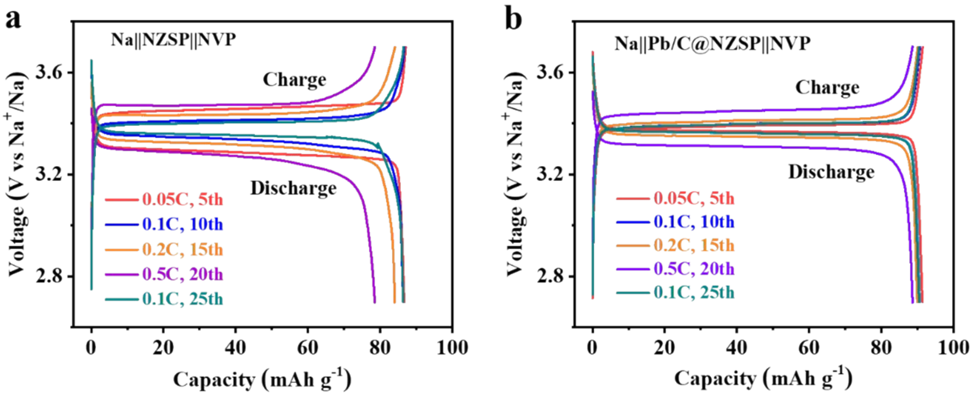

Figure S12. The charge/discharge curves of (**a**) Na||NZSP||NVP and (**b**) Na||Pb/C@NZSP||NVP cells for the last cycle of 0.05C, 0.1C, 0.2C, 0.5C and 0.1C during rate capability tests.

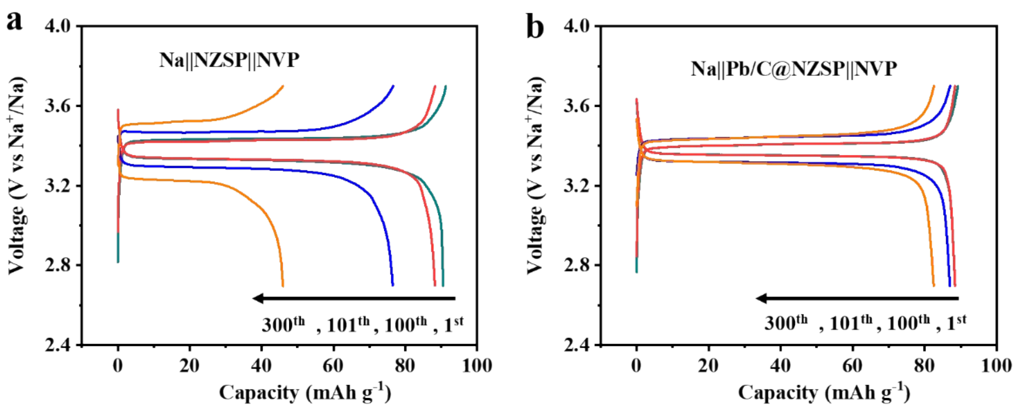

Figure S13. The charge/discharge curves of (**a**) Na||NZSP||NVP and (**b**) Na||Pb/C@NZSP||NVP cells for the initial and last cycle at 0.2C (100 cycles) and 0.5C (200 cycles).

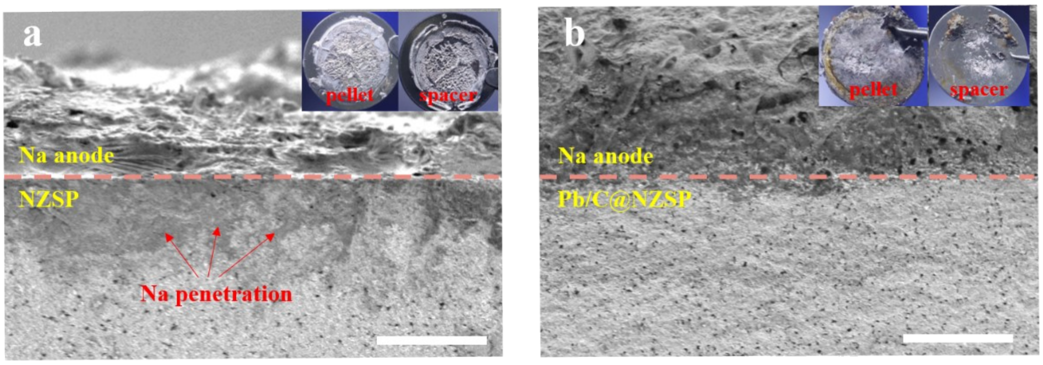

Figure S14. Postmortem SEM characterizations conducted on the anode interfaces of the cycled (**a**) Na||NZSP||NVP and (**b**) Na||Pb/C@NZSP||NVP cells. Insets show the corresponding digital

images taken on both pellet and spacer sides. Scale bars: 100 μm.

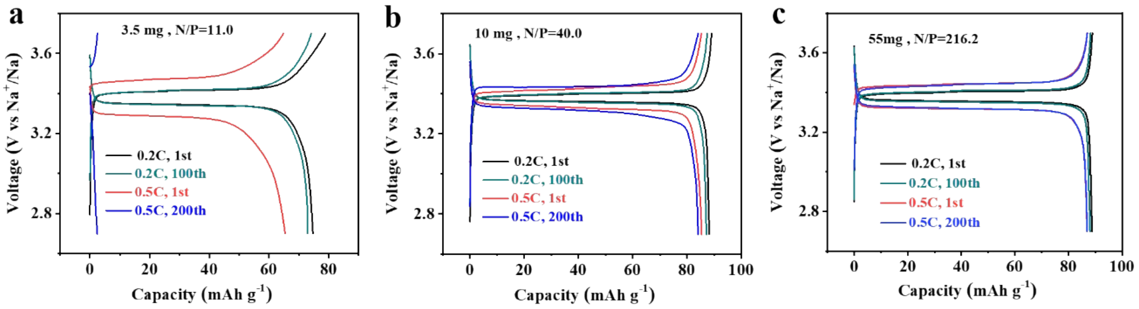

Figure S15. The charge/discharge curves of Na||Pb/C@NZSP||NVP cells with different Na loadings, or various N/P ratios: (**a**) N/P=11.0, (**b**) N/P=40.0, and (**c**) N/P=216.2.

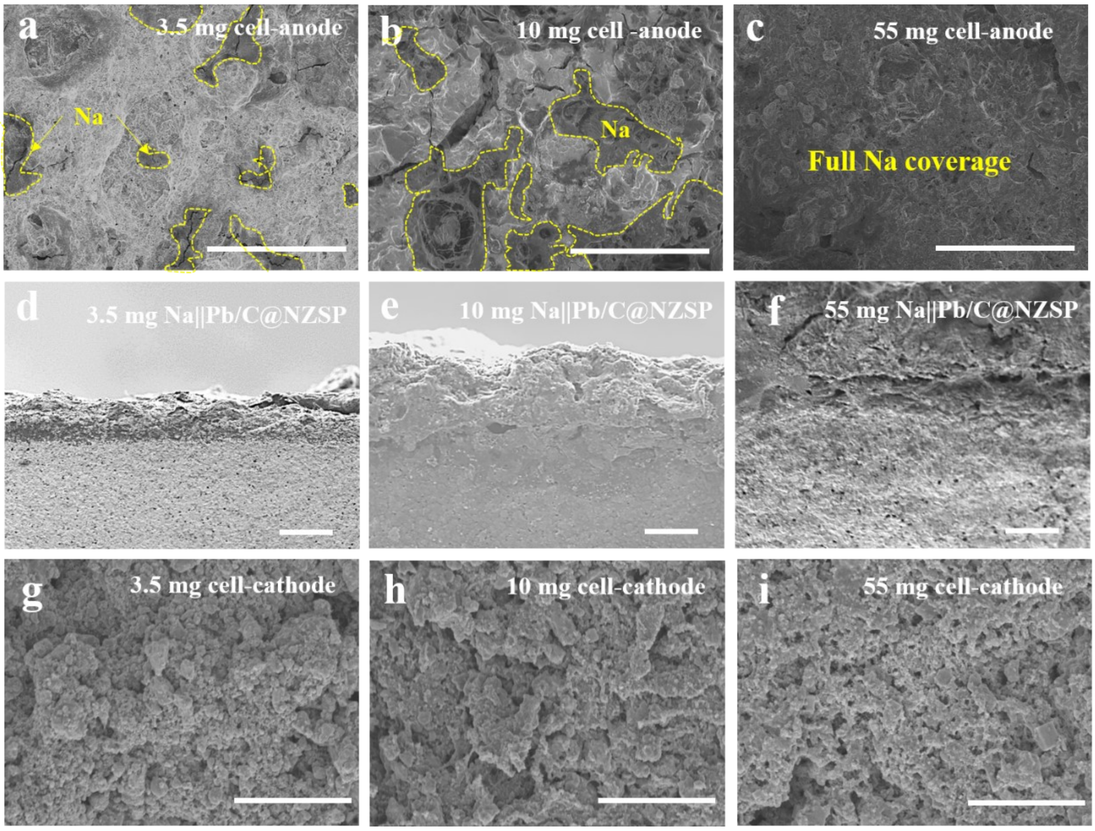

Figure S16. Postmortem SEM characterizations conducted on the (**a-c**) Na anode surfaces, (**d-f**) Na||Pb/C@NZSP cross-sections and (**g-i**) NVP cathode surfaces of the cycled Na||Pb/C@NZSP||NVP cells loaded with (**a, d, g**) 3.5, (**b, e, h**) 10, and (**c, f, i**) 55 mg Na. Scale

bars: 100 μm for **a-f** and 5 μm for **g-i**.

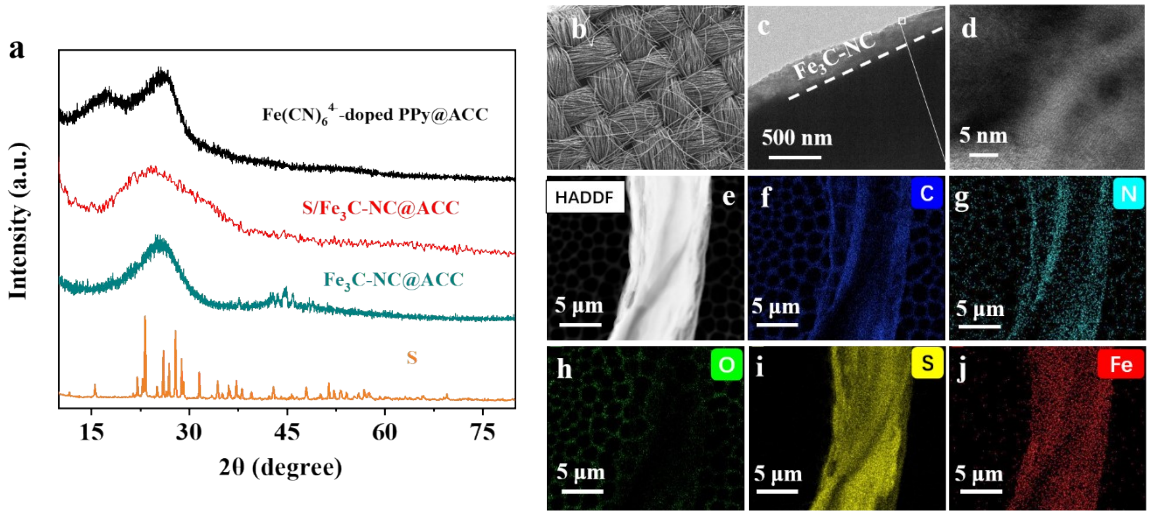

Figure S17. (**a**) XRD diffraction patterns of S, Fe_3C-NC@ACC, S/Fe_3C-NC@ACC and Fe(CN)$^{4-}$-doped PPy@ACC. (**b**) SEM, (**c-d**) HAADF-STEM images and (**e-j**) the corresponding EDX mappings of S/Fe_3C-NC@ACC.

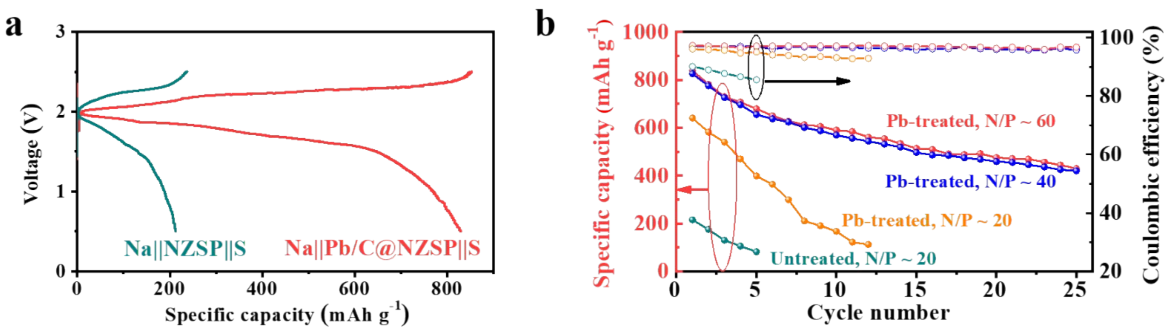

Figure S18. (**a**) The 1st charge/discharge curves of Na||NZSP||S and Na||Pb/C@NZSP||S batteries. (**d**) The cycling performances of Na||Pb/C@NZSP||S batteries under different N/P ratios of 20, 40, and 60.

Table S1. The fitting results of EIS spectra acquired on different Na symmetric cells.

Symmetric cell	R_b (ohm)	R_{gb} (ohm)	C_{gb} (F)	R_{int} (ohm cm^2)	C_{int} (F)
NZSP, 0 MPa	51.86	120.6	5.53×10^{-9}	391	5.15×10^{-8}
NZSP, 15 MPa	85.69	80.94	9.53×10^{-10}	87.5	3.07×10^{-8}
Pb/C@NZSP	81.57	88.86	9.19×10^{-10}	1.5*	/

***Note:** The effective area used for calculating ASR_{int} is 0.8 cm^2. For the Na||Pb/C@NZSP||Na cell, the grain boundary and interface contribution cannot be separated as shown in Fig. 3b. Therefore, the $R_{int.}$ of 3.72 Ω was estimated by subtracting the total cell resistance (R_b+ R_{gb}) of 170.43 Ω by the total resistance of the NZSP pellet (166.71 Ω, **Fig. S1**).

Table S2. Fitted cell resistances from EIS analysis of full cells shown in Fig. 5d-f.

Cell notation	R_{Cell}, initial (Ω)	R_{Cell}, after 0.2C (Ω)	R_{Cell}, after 0.5C (Ω)
3.5 mg	321.7	413.7	675.3
10 mg	207.7	228.6	308.0
55 mg	262.4	349.1	434.5

Table S3. Summary of parameters of different interfaces for DFT calculations.

Interfaces	No. of Atoms	a/Å	b/Å	W_{ad}/J m^{-2}
Na (001)/ NZSP (001)	192	9.1187	18.2388	0.25
Na (001)/ Pb (001)	292	20.2485	20.2485	0.44
Na (001)/ Na_2CO_3 (001)	160	18.1879	5.3007	0.12
Na (001)/ C (001)	92	4.2608	12.3000	0.08

References

[1] K. Lu, H. Zhang, S. Gao, H. Ma, J. Chen, Y. Cheng, *Advanced Functional Materials* **2019**, 29, 1807309.

[2] Kresse, Furthmuller, Physical review. B, *Condensed matter* **1996**, 54, 11169.

[3] Perdew, Burke, Ernzerhof, *Physical review letters* **1996**, 77, 3865.

[4] J. P. Perdew, J. A. Chevary, S. H. Vosko, K. A. Jackson, M. R. Pederson, D. J. Singh, C. Fiolhais, *Phys Rev B Condens Matter* **1992**, 46, 6671.
